# Evaluation of vehicle running performance on ash-covered roads

**DOI:** 10.1038/s41598-023-47122-8

**Published:** 2023-12-06

**Authors:** Tatsuji Nishizawa, Mitsuhiro Yoshimoto, Tomohiro Kubo, Ryo Honda, Setsuya Nakada, Nobuko Kametani, Yasuhiro Ishimine, Shinya Yamamoto

**Affiliations:** 1 Mount Fuji Volcanic Disaster Research Center, Mount Fuji Research Institute, Yamanashi Prefectural Government, Yamanashi, Japan; 2 Center for Integrated Volcano Research, National Research Institute for Earth Science and Disaster Resilience, Ibaraki, Japan

**Keywords:** Natural hazards, Volcanology

## Abstract

The scale of volcanic eruptions influences the effects of wide-ranging disasters involving ashfall, such as the disruption of lifelines and paralysis of urban functions. This highlights the importance of vehicle availability on ash-covered roads for disaster management personnel involved in rescue and recovery efforts and for citizens who must evacuate or continue their social lives. We conducted tests to scientifically verify the running ability of vehicles on ash-covered roads. Results revealed that all-wheel-drive vehicles showed better running performance than two-wheel-drive vehicles, which get stuck when ashfall thickness exceeds 10 cm. Most of the vehicle’s drive power is consumed as energy to scrape ash grains from under the tires, hindering sufficient propulsion. In addition, the tires sink into the ash layer, which increases driving resistance and causes the vehicle to get stuck. Running ability on ash-covered roads is mainly determined by the relation between the “drive system” of the vehicle and the “thickness of ash” on the roads. In addition, road surface conditions, including ash thickness, could change in time and space because of traffic volume and weather conditions.

## Introduction

Compared with earthquakes, tsunamis, and floods, volcanic eruptions are characterized by their low occurrence frequency, long duration, and diverse factors, which make disaster response difficult. For example, since the sixteenth century, approximately 1.3 volcanic eruptions have occurred annually, with 56% of these resulting in fewer than 10 fatalities^1^. Between 1995 and 2004, the total number of victims of volcanic eruptions worldwide was approximately 2% of those of earthquakes and tsunamis^2^. Volcanic disasters include direct and secondary damages; the former is caused by volcanic ejecta such as lava and pyroclastic flows and ballistic ejects, and the latter includes rainfall-triggered debris flows after an eruption. Volcanic ejecta vary in temperature, moving speed, and direction depending on their type. Furthermore, areas of influence differ depending on the crater location, the scale (or explosivity), the eruption style, and the season. This problem complicates the decision-making process regarding when, where, and how to evacuate.

Volcanic plume, a popular eruptive phenomenon, is a gas–solid multiphase fluid composed of a mixture of volcanic ejecta and the atmosphere. In explosive volcanic eruptions, pyroclastic materials are ejected from the crater in high-speed jets with volcanic gas. When the ejecta takes in a large amount of air, it becomes lighter than the atmosphere and rises buoyantly, forming an ash plume^3^. The plume expands horizontally, reaching air altitude that balances its atmospheric density, and its behavior depends on the eruption scale and wind strength^4, 5^. The volcanic gas in the smoke diffuses into the atmosphere, and pyroclastic particles such as scoria, pumice, and volcanic ash eventually fall to downwind areas (hereafter called ashfall). The thickness and grain size of the fallen ash become thinner and finer the further it is from the crater and the main volcanic plume axis^3, 6^. Ashfall spreads to a wide area, reaching hundreds of kilometers depending on the eruption scale, but volcanic disasters cause relatively few human casualties. Meanwhile, lifelines such as electricity, water, and communication networks could be disrupted, paralyzing urban functions and considerably affecting society. Depending on the thickness (or amount) of ashfall, the collapse of old wooden houses and debris flows increase the risk of human casualties^6^.

Ashfall on roads may significantly influence transportation, living, and social and industrial infrastructure as well as residents’ evacuation actions. Many obstacles, such as poor visibility, make it almost impossible to drive vehicles safely during ashfall^7^. Regardless of the thickness of the ashfall, roads should not be opened to vehicle traffic until the ash is completely removed and road safety is ensured. Meanwhile, disaster prevention organizations, which must urgently conduct disaster response, restoration, and rescue operations, may be forced to dispatch support vehicles before ash removal is completed on the roads. In addition, the wider the scope of traffic restrictions associated with ash removal work and the slower the restoration efforts, the more significant the social impact and economic loss. Therefore, the national government, local governments, and private companies with jurisdiction over roads in areas where ashfall is expected must create facilities and systems for prompt ash removal operations. However, there have been a few cases of scientific verification based on running tests aiming to answer the fundamental question: “How far can vehicles run on ash-covered roads?”^8, 9^. At least in Japan, no attempts have been made among disaster prevention officials and residents in areas at risk of ashfall to drive a vehicle in a simulated post-ashfall environment, which is essential for discussing and drafting evacuation plans and response measures in an eruption disaster.

In 2021, the hazard map of Mt. Fuji, the highest and largest active volcano in Japan, was revised for the first time in 17 years^10^. According to the 2004 version of the ashfall hazard map^11^, which was not revised in 2021, an explosive eruption of the same magnitude as the Hoei eruption is expected to produce volcanic ash up to 10 cm thick in Tokyo, which has a population of 14 million and is located approximately 100 km east of Mt. Fuji. The Hoei eruption, the most recent of which took place in 1707, was a Plinian eruption, which is unusual for Mt. Fuji, mainly composed of basaltic magma, with an estimated eruption volume of 0.7 km dense rock equivalent^12–14^.

Yamanashi Prefecture, which covers the northern half of Mt. Fuji, held a “Driving experience events on roads covered with ash fall deposits” in November 2021 to raise awareness about the revised hazard map. Here, residents and disaster prevention officials drove vehicles on courses simulating various road conditions after ashfall. Residents drove front-wheel-drive (FWD) commercial vehicles prepared by the organizer. In Japan, approximately 80% of passenger cars owned by ordinary households are two-wheel-drive vehicles^15^. The participants experienced a difference in driving sensation from paved roads and the difficulty of driving in such conditions. The purpose was to discourage residents from driving in emergencies because if they insist on driving on ash-covered roads, they may cause traffic congestion, delay evacuation due to frequent accidents, and interfere with emergency vehicles responding to the disaster.

Meanwhile, disaster prevention personnel drove the courses using official vehicles, such as firetrucks, police vehicles, and Self-Defense Forces vehicles, which are expected to be deployed in an emergency. The aim was for staff to understand the vehicles’ running performance and recognize precautions when driving on ash-covered roads. The three-day event involved approximately 170 residents and 70 disaster prevention organizations^16^.

Two years later, in conjunction with the revision of the hazard map, the Fuji Volcano Evacuation Basic Plan was published^17^, which outlined the timing and means of evacuation for residents in the foot area for each eruption and human attribute. In addition, the plan stipulated that when an ashfall occurs, the primary rule is to evacuate indoors, which reflects the insights obtained from driving events. Japan has several other active volcanoes, such as Sakurajima and Aso, that are adjacent to cities. The national and local governments are beginning to discuss measures to respond to wide-area ash fall^6^. Meanwhile, many countries and regions with active volcanoes, including Japan, have no guidelines regarding the use of ash-covered roads.

The development of appropriate traffic regulations during volcanic eruptions with ashfall requires a quantitative and scientific confirmation of vehicle running performance on ash-covered roads based on tests. To this end, we conducted dynamic testing on courses to obtain basic knowledge of vehicles’ running ability on ash-covered roads. In these tests, we focused on the following foundational aspects: running, braking, and turning. We then reported our findings on these running tests, which verified the distance that the vehicles could travel while on ash-covered roads.

## Results

### Start and sudden-start test

**Figure 1:**
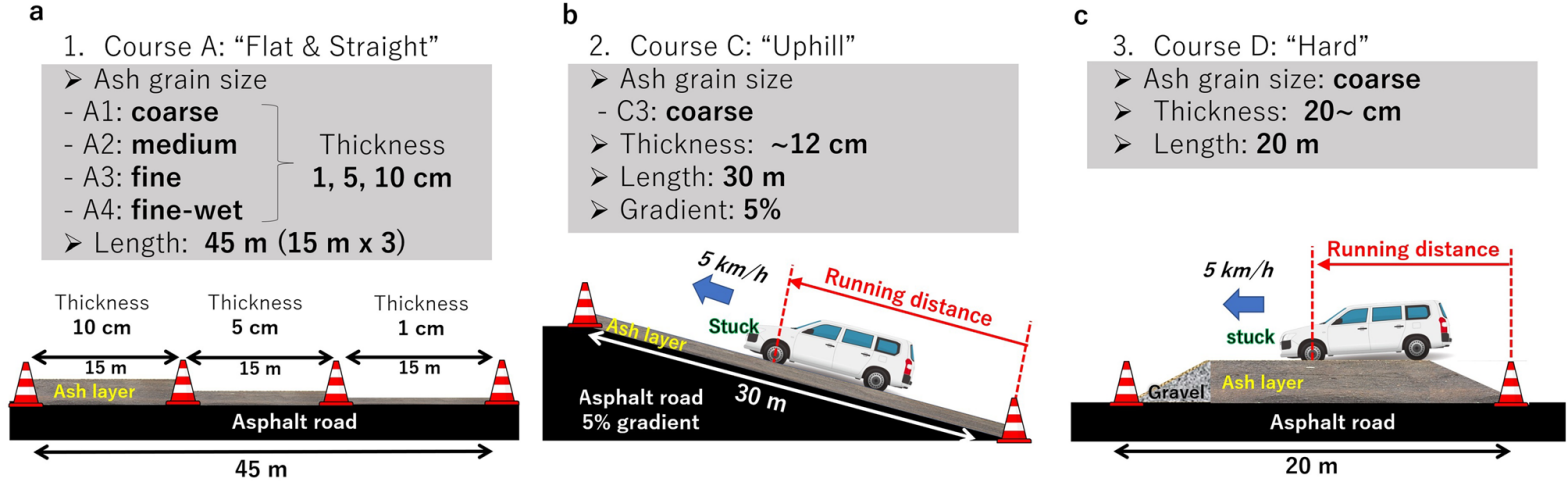
Test courses and methods. (**a**) Course A has four tracks consisting of 45 m long flat paved roads covered with coarse-, medium-, fine-, or wetted fine-grained ash at 1 cm, 5 cm, and 10 cm thickness every 15 m length. (**b**) Course C is uphill. Course C3 is 30 m long and 5% uphill paved roads covered with coarse-grained ash at approximately 12 cm thickness. (**c**) Course D consists of 20 m long flat paved roads covered with coarse-grained ash over 20 cm thick, which is difficult for vehicles to run through.

This test was conducted on four flat courses on A (A1–A4) and one of the four uphill courses on C (C3) (Fig. 1, Table S1). In flat course A, all test vehicles were able to start and sudden-start with no issues, but differences in behavior and stability were observed depending on the drive system. With increasing ash thickness, two-wheel-drive vehicles tended to slip and lose steering wheel control during sudden starts. Especially for rear-wheel-drive (RWD) vehicles, the steering angle needed to be corrected to drive straight because of the lateral slip of the vehicle’s rear. Test vehicles equipped with traction control could control slippage by activating the system during sudden start. The wet fine-grained volcanic ash road surface (A4) was more slippery than the dry ash grain (A3), and an increase in slip rate was observed. Meanwhile, the all-wheel-drive (AWD) vehicles showed stability without slipping during both start and sudden start. Similarly, the AWD vehicles were stable from start to finish even when starting or sudden-starting on the 5% uphill slope of C3. No start and sudden-start tests were performed for two-wheel-drive vehicles on courses C3 and D as these all got stuck on the courses (see Methods section for details).

### Passing test

**Figure 2:**
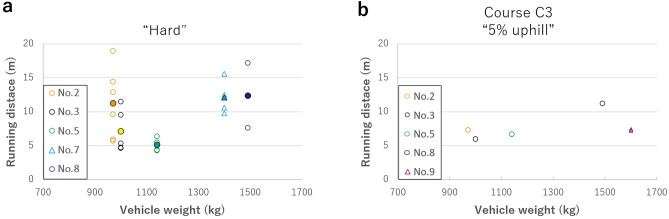
Results of passing tests for two-wheel-drive vehicles. Running distances on (**a**) course D and (**b**) course C3. The horizontal axis is the vehicle weight, and the vertical axis is the running distance. Circles indicate FWD vehicles, triangles indicate RWD vehicles, and fills indicate average values.

**Figure 3:**
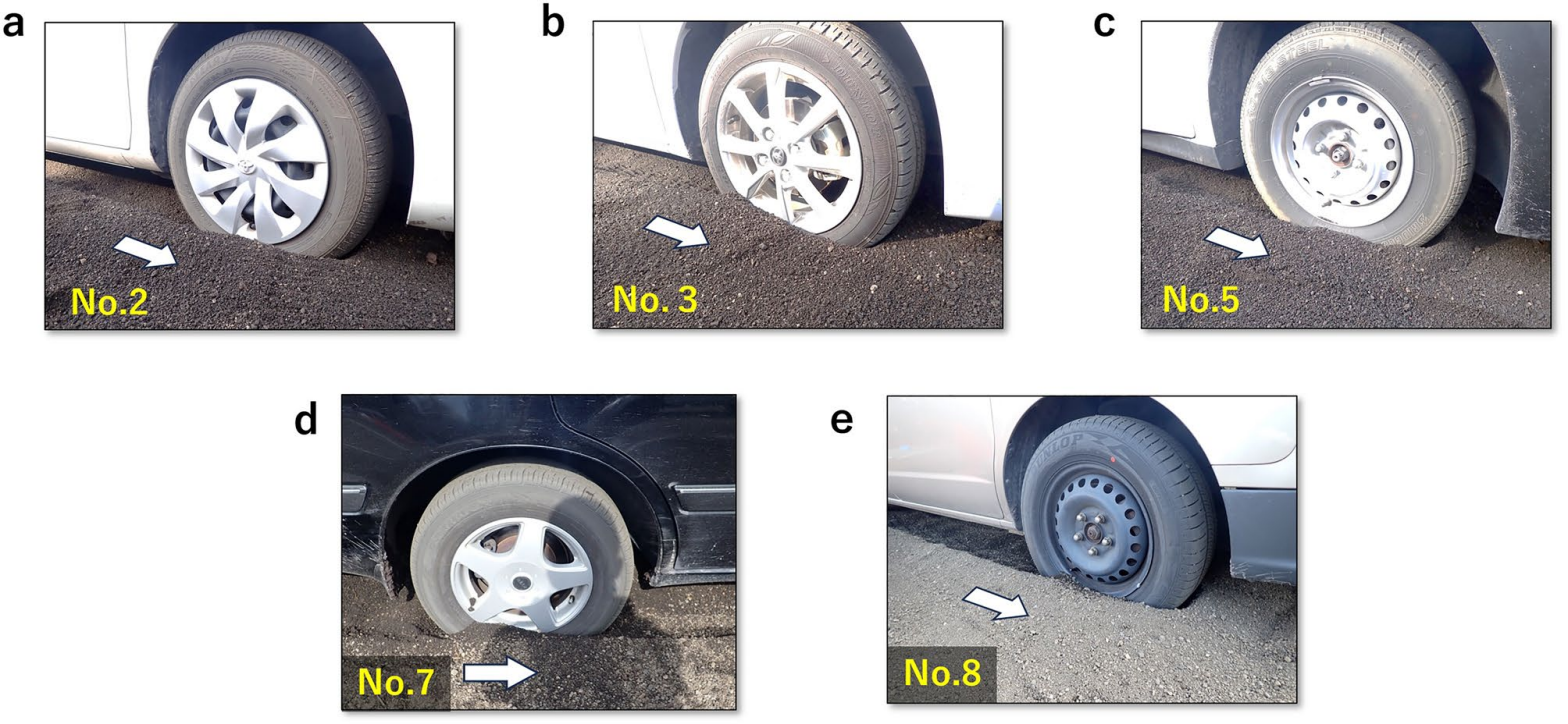
Degree to which the drive wheels of two-wheel-drive vehicles sink into the ash-covered roads when stuck. Vehicles get stuck when half the height of the tires sink into the ash layer. Test vehicles are (**a**) No. 1 (FWD, 970 kg), (**b**) No. 3 (FWD, 1000 kg), (**c**) No. 5 (FWD, 1140 kg), (**d**) No. 7 (RWD, 1400 kg), and (**e**) No. 8 (FWD, 1490 kg). The white arrows indicate the vehicle's running direction.

In most cases, the two-wheel-drive vehicles were stuck on hard course D and uphill course C3, making it impossible to pass through (Fig. 2, Table 1). As the test vehicle entered the course, the drive wheels gradually slipped and scraped out the ash grains, making it difficult to maintain vehicle speed. Eventually, the tires sank into the ash grain layer and got stuck (Fig. 3). Running distance varied widely among vehicles: approximately 5–13 m on course D and approximately 6–7 m on course C3, with no correlation observed between vehicle weight and whether the vehicle was equipped with an FWD or RWD system (Fig. 2, Tables S2, S3). However, for ashfall thickness below 10 cm, two-wheel-drive vehicles could pass on the flat and uphill courses. AWD vehicles could pass stably with little or no slippage. Simply put, the vehicles’ running ability on ash-covered roads depended on their drive system (Fig. 2, Table 1). Because similar results were obtained with different drivers in the same test, the results are universal for average drivers.

**Table 1:**
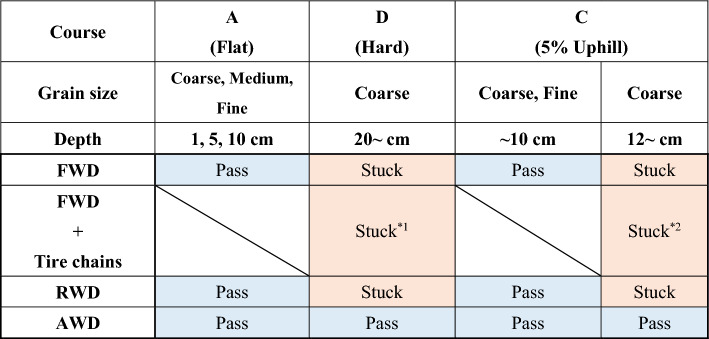
Summary of the passing tests. *1 The same results with or without tire chains. *2 The running distances are reduced by 20%–40% compared to when tire chains are not installed.

**Figure 4:**
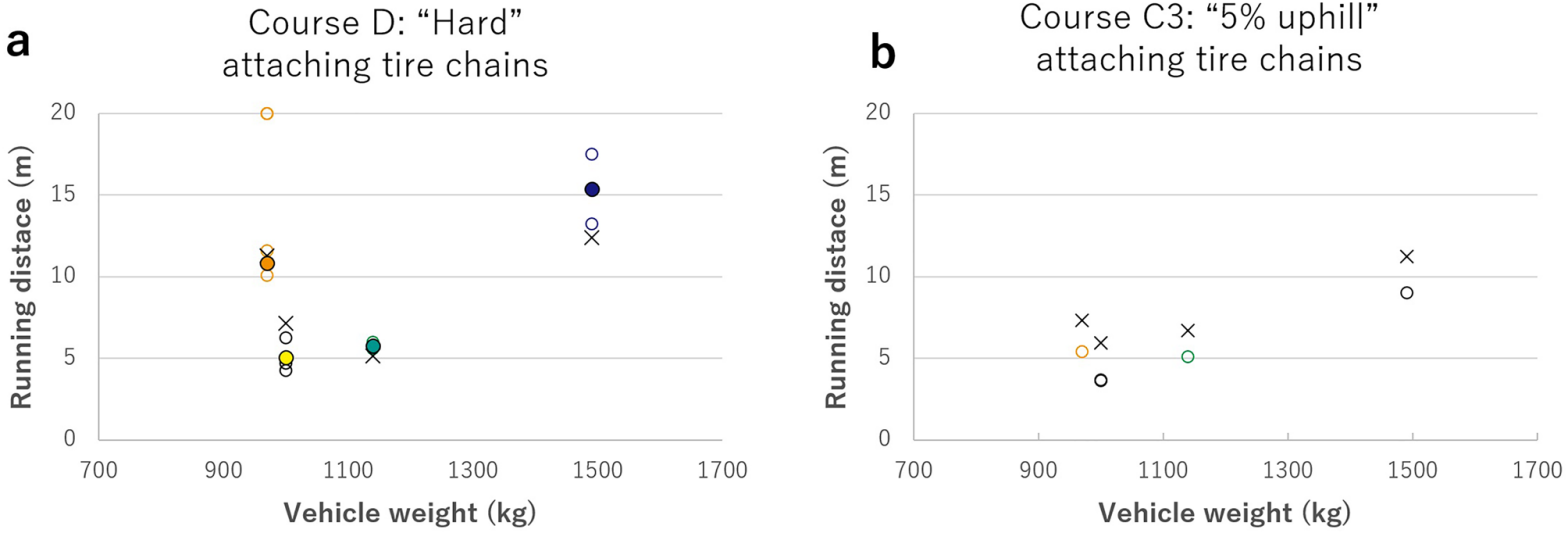
Results of passing tests for two-wheel-drive vehicles equipped with tire chains. Running distances on (**a**) course D and (**b**) course C3. The horizontal axis is the vehicle weight, and the vertical axis is the running distance. Cross marks signify the average distance without tire chains. The other symbols are the same as in Fig. 2.

The same test was performed by attaching tire chains, which are effective in improving safety when driving on snowy roads, to the driving wheels of the stuck FWD vehicles (Fig. S1) to examine their effect on driving performance on ash-covered roads. Results showed no increase in running distance from the previous test on courses D and C3 (Fig. 4). The drive wheels began to slip as well, and the tires sunk into the ash layer and got stuck. On course D, no significant change in running distance was observed. On course C3, the driving wheels spun earlier than when the tire chains were not installed, and running distance decreased by 20–40% (1.6–2.2 m).

As described above, vehicle stability and running performance on ash-covered roads depend mainly on the drive system, with AWD vehicles displaying higher stability and running performance than two-wheel-drive vehicles. Under this test’s conditions, the ash thickness threshold at which two-wheel-drive vehicles can travel is approximately 10 cm. Meanwhile, AWD vehicles could run on a flat course with 20 cm thick ash and a 5% uphill with 12 cm thick ash. In addition, the installation of tire chains was deemed inconsequential to the improvement of vehicle running ability. Moreover, ash-covered roads are more slippery than paved roads. On ash-covered roads, the thicker the ashfall, the harder it is to control the vehicle and the greater the running resistance, so the opening of the accelerator pedal must be increased to maintain speed.

## Discussion

We proposed a physical model in which a two-wheel-drive vehicle traveling on a road with ashfall gets stuck based on the test results and provided photo and video materials during the tests.

### Factors that cause vehicles to get stuck on ashfall roads

**Figure 5:**
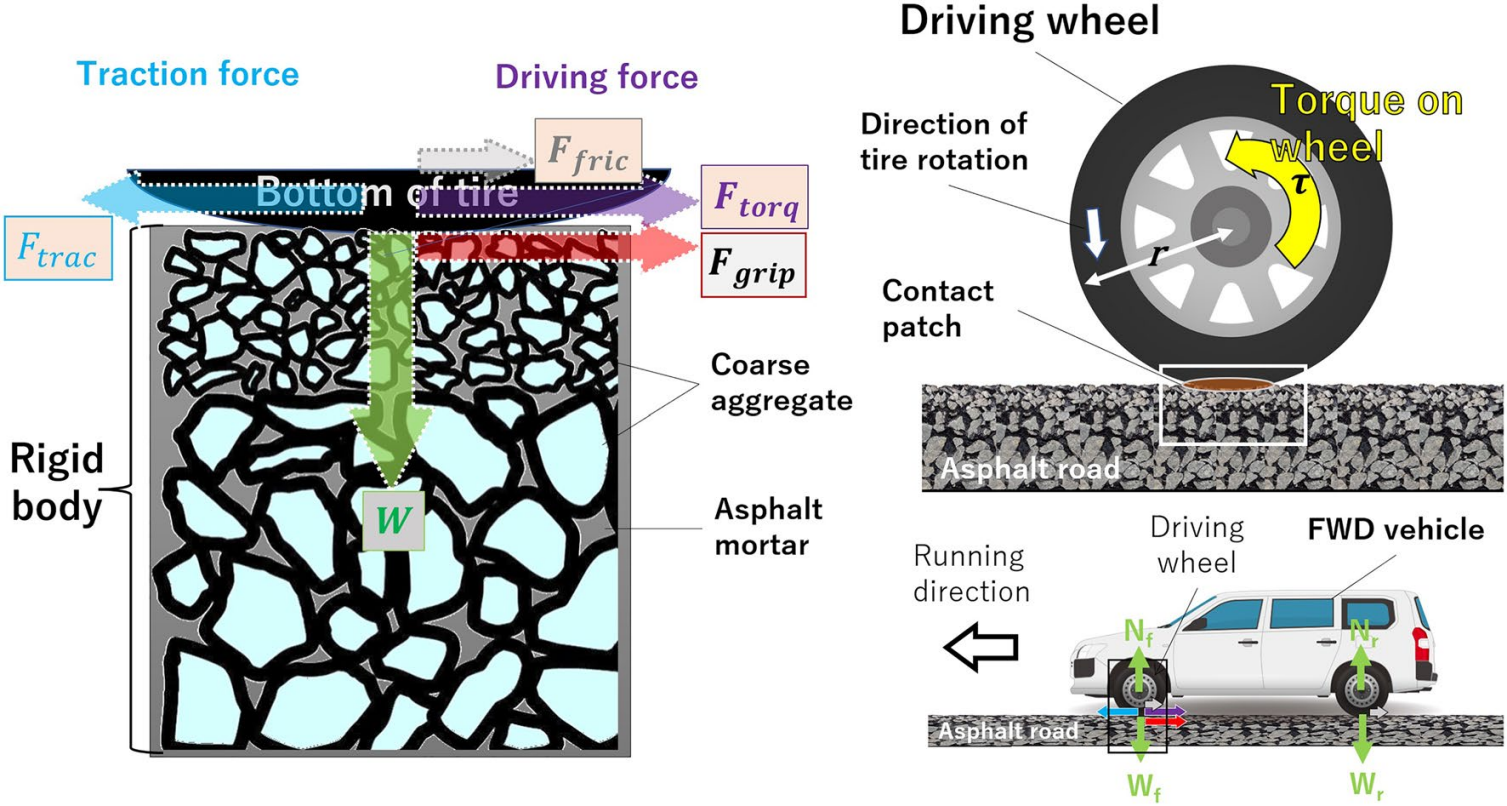
Forces acting between the drive wheels and the road surface when an FWD vehicle runs on flat paved roads. The lower right area shows the whole vehicle, the upper right area is an enlarged view of the drive wheels, and the left is an enlarged view of the contact area between the tires and the road surface. The brown oval with gray edge indicates the contact patch between the tire and the paved roads. See text for details on each of these forces. The figure is not drawn to scale. The cross-sectional view of the asphalt road was taken from Wikimedia Commons (file: Doorsnede ZOAB.jpg).

Figure 5 shows the forces acting on a vehicle traveling on a paved road. First, the wheel pushing the ground exerts a vertical downward force “W”, and then the ground supporting the wheel exerts a vertical upward force “N”, which is equal to the vehicle weight distributed to each wheel (half of the front or rear axle weight). Next, the component of the force acting horizontally is given.

The torque generated by the engine is first decelerated by the transmission, and the force that drives the tires of the drive wheels ($F_{torq}$) works in the opposite direction of the vehicle’s travel on the contact surface with the road as shown in the following equation:

1 $$\begin{array}{*{20}c} {\tau = T_{e} \cdot \rho n \cdot \rho F \cdot \eta n \cdot \eta F} \\ \end{array}$$

2 $$\begin{array}{*{20}c} {F_{torq} = {\raise0.7ex\hbox{$\tau $} \!\mathord{\left/ {\vphantom {\tau r}}\right.\kern-0pt} \!\lower0.7ex\hbox{$r$}}} \\ \end{array}$$

where $\tau$ is the torque acting on the drive wheels, $T_{e}$ is the engine torque, $\rho n$ is the transmission gear ratio, $\rho F$ is the final gear ratio, $\eta n$ is the transmission transfer efficiency, $\eta F$ is the final reduction transfer efficiency, *F*_*torq*_ is the force at the edge of the wheel, and $r$ is the radius or distance from the center. When a vehicle runs at a constant speed on a flat road, $F_{torq}$ does not change.

Then, the grip force of the tire on the road surface ($F_{grip}$) is expressed by the following:

3 $$\begin{array}{*{20}c} {F_{grip} = \mu \cdot W} \\ \end{array}$$

where $\mu$ is the coefficient of the friction between the road surface and tire, and $W$ is the vehicle weight distributed to the wheel.

As a force counteracting the action force of the driving wheels kicking the road surface, the propulsive force with which the road surface kicks the driving wheels ($F_{trac}$) is generated in the opposite direction. Rolling resistance $F_{fric}$, which consists of deformation, ground friction, and air resistance, is generated as running resistance.

4 $$\begin{array}{*{20}c} {F_{fric} = C_{r} \cdot W} \\ \end{array}$$

where $C_{r}$ is the rolling resistance coefficient.

Next, we consider the principle of vehicles getting stuck on ash-covered roads. Just before getting stuck, the drive wheels of two-wheel-drive vehicles begin to slip (the vehicle moves slower relative to the rotation speed of the drive wheels), and the tires sink into the ash layer on the road (Fig. 3). Tire slipping takes place when, $F_{torq}$ becomes greater than $F_{grip}$, the force at which the tire grips the road surface.

5 $$\begin{array}{*{20}c} {F_{torq} > F_{grip} } \\ \end{array}$$

Meanwhile, the following equation describes the condition in which tires do not slip^18^.

6 $$\begin{array}{*{20}c} {F_{torq} \leqq F_{grip} } \\ \end{array}$$

Asphalt mixtures, which are widely used for pavement surfaces, involve the mixing and bonding of aggregate and asphalt (adhesive: binder) at a high temperature so that the pavement surface behaves as a rigid body (Fig. 5) and is therefore not deformed by the vertical or horizontal forces acting on the tires.

**Figure 6:**
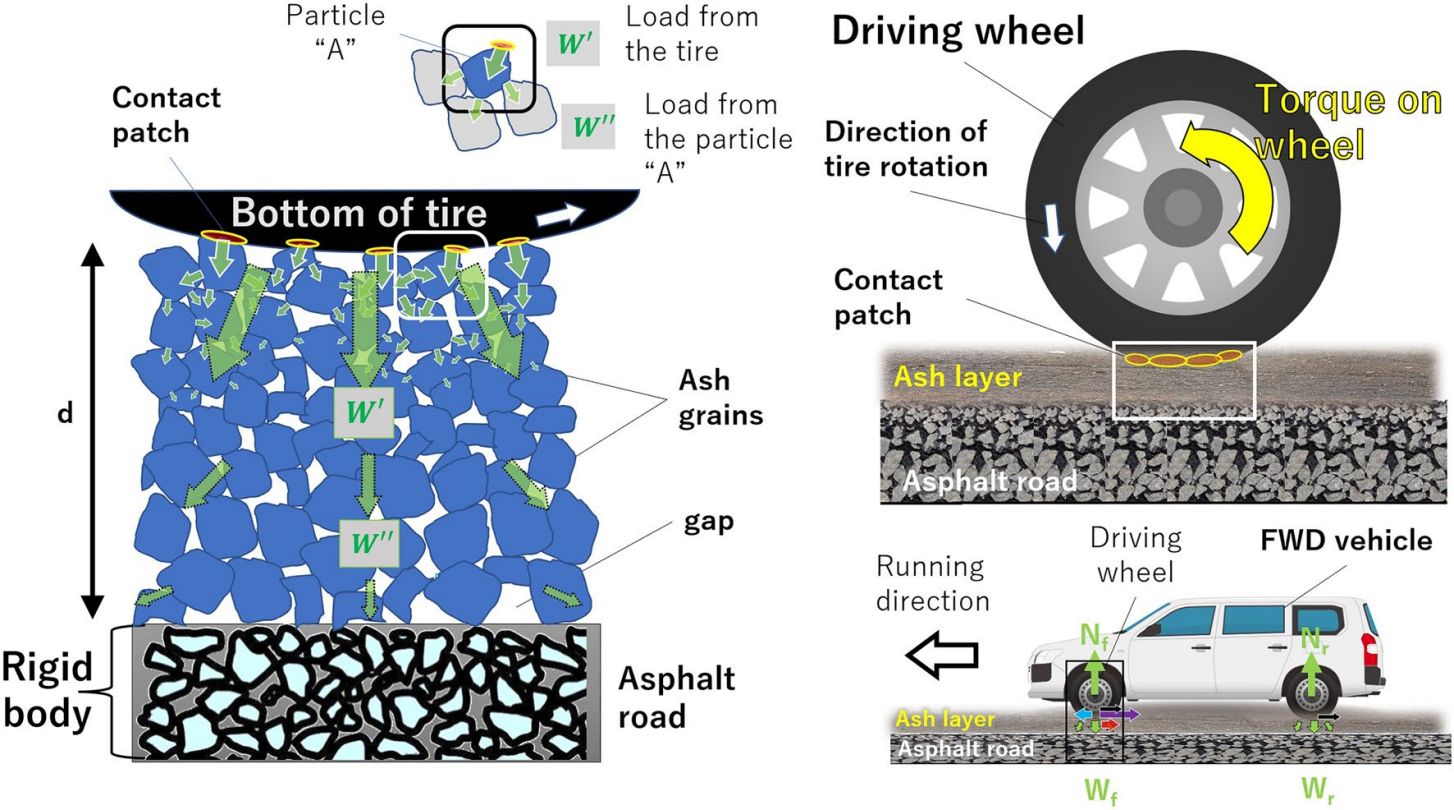
Vertical forces acting between the drive wheels and the ash layer when an FWD vehicle runs on ash-covered roads. The lower right part shows the whole vehicle. The upper right part is an enlarged view of the drive wheels. The left is an enlarged view of the contact area between the tire and the ash layer. The light-green arrows indicate vehicle loads acting on the ash grains. The upper left is an enlarged view of an ash grain “A” and the forces acting on it. The brown ovals with yellow edges indicate the contact patch between the tire and the ash grains. See text for details on each of these forces. “d” pertains to ash fall thickness. The figure is not drawn to scale. The cross-sectional view of the asphalt road was taken from Wikimedia Commons (file: Doorsnede ZOAB.jpg).

Ash-covered roads, meanwhile, are composed of volcanic ash grain deposits on paved road surfaces, where these grains are not bonded to each other, with gaps between grains (Fig. 6). When a vehicle travels on these roads, its weight acting vertically downward is distributed to the ash grains in contact with the tread (*W′*), which is then dispersed to other grains in contact with those grains (*W″*). The vehicle’s weight is distributed downward within the ash deposition layer. When this load causes a breakdown and/or displacement of ash grains, the grain size and the gap decrease, and ruts are formed. On paved roads, aggregates are bonded to each other by asphalt, so particles do not deform and are not displaced, and the wheels do not sink into the road (Fig. 5).

**Figure 7:**
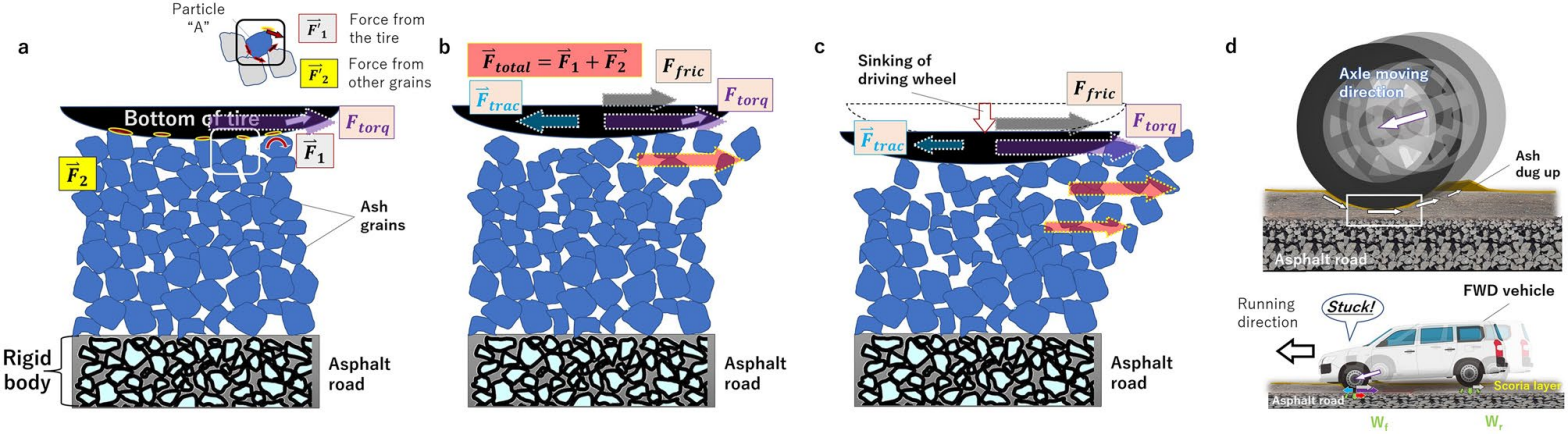
Horizontal forces acting between the drive wheels and the ash layer when an FWD vehicle runs on ash-covered roads. (**a**) Force $\vec{F}_{1}$ acts on the ash grains in contact with the tire rotating at torque ($\vec{F}_{torq}$). Force $\vec{F}_{2}$, meanwhile, is the shear stress that the grains in contact with the tire receive from the other grains. $\vec{F}_{2}$ acts in the opposite direction to $\vec{F}_{1}$. (**b**) Then, the magnitude of $\vec{F}_{1}$ is larger than that of $\vec{F}_{2}$, and the combined force ($\vec{F}_{Total} = \vec{F}_{1} + \vec{F}_{2}$) causes the grains to move in the direction of the tire rotation. (**c**) The grains under the tire move backward, and the tires sink into the ash layer. The vehicle then gets stuck because of increased running resistance as the ash grains in front of the tires increase. (**d**) The drive axle trajectory (upper figure) and the whole vehicle (lower figure) when it gets stuck. The figure is not drawn to scale. The cross-sectional view of the asphalt road was taken from Wikimedia Commons (file: Doorsnede ZOAB.jpg).

Similarly, the forces driving the tire $$F_{torq}$$ are transmitted to the ash grains in contact with the tread (Fig. 7a). Focusing on a single ash grain “A” in contact with the tread, this force generates a moment $$\overset{\lower0.5em\hbox{$\smash{\scriptscriptstyle\rightharpoonup}$}}{F^{\prime}}_{1}$$ that rotates the grain (clockwise in Fig. 7a). The magnitude of this force depends on $$F_{torq}$$, *W′*, the friction coefficient between the tire and the ash grains, the shape of the ash grains, and the tire’s tread pattern. For simplicity, the tread pattern is ignored here (alternatively, the same would be true if the ash grain size were sufficiently large relative to the width of the grooves in the tread pattern). Next, from other grains in contact with particle A, a resistant force $$\overset{\lower0.5em\hbox{$\smash{\scriptscriptstyle\rightharpoonup}$}}{F^{\prime}}_{2}$$ acts in the opposite direction to stop the particle’s rotational moment. The magnitude of this force depends on $$F_{torq}$$, $$W^{{\prime \prime }}$$, the shape of the ash grains, and the friction coefficient between ash grains (Fig. 7a). The total $$\overset{\lower0.5em\hbox{$\smash{\scriptscriptstyle\rightharpoonup}$}}{F^{\prime}}_{1}$$ forces acting on all grains in contact with the tire can be interpreted as the force $$\overset{\lower0.5em\hbox{$\smash{\scriptscriptstyle\rightharpoonup}$}}{F}_{1}$$ pushing these grains in the direction of the tire rotation. Meanwhile, the sum of $$\overset{\lower0.5em\hbox{$\smash{\scriptscriptstyle\rightharpoonup}$}}{F^{\prime}}_{2}$$ can be interpreted as the shear stress $$\overset{\lower0.5em\hbox{$\smash{\scriptscriptstyle\rightharpoonup}$}}{F}_{2}$$ with respect to $$\overset{\lower0.5em\hbox{$\smash{\scriptscriptstyle\rightharpoonup}$}}{F}_{1}$$ (Fig. 7a).

All tires of the drive wheels of the stuck two-wheel-drive vehicles had sunk into the ash layer, indicating that the ash grains under the treads were crushed by the vehicle weight acting downward and moved backward by the drive wheel rotation (Fig. 3). In this case, $$F_{torq}$$ is consumed as energy to move the ash grains in contact with the tread backward, causing the drive wheel to slip approximately, and the actual $$F_{trac}$$ obtained becomes smaller (Fig. 7b). The drive wheels dig out the grains beneath them and therefore sink into the ash layer, while the increased ash grains in front of the drive wheels increase driving resistance, eventually causing the vehicle to get stuck (Fig. 7c, d). In addition, with the increase in ash thickness, the vehicle weight distribution rate acting vertically downward increases as well (Fig. 6), which means that the force (*W”*) that presses ash grains against each other decreases with distance from the tread, so the magnitude of $$\overset{\lower0.5em\hbox{$\smash{\scriptscriptstyle\rightharpoonup}$}}{F}_{2}$$ relative to $$\overset{\lower0.5em\hbox{$\smash{\scriptscriptstyle\rightharpoonup}$}}{F}_{1}$$ is reduced. Simply put, the thicker the ash layer on the road, the higher the probability of a vehicle getting stuck.

### The effect of installing tire chains and the differences between ash-covered roads and snowy and icy roads or gravel roads

Tire chains did not improve the vehicle’s running distance on ash-covered roads (Fig. 4). The factors are considered based on the model proposed above. The main reason is that the unevenness of the contact surface between the tire and road surface and the power to scrape out the ash grains underneath have increased. Slipping and getting stuck on ash-covered roads are fundamentally different from when they take place on icy or snow-covered roads. Installing tire chains increases the grip between the tires and the surface of icy or snow-covered roads, thereby improving safety and running performance. Since icy or compacted snow roads behave as rigid bodies, little deformation occurs in response to force inputs from the drive wheels. The coefficient of friction between the tire and ice or snow is approximately 7–40% or less than that for paved roads, so the force $F_{grip}$ is significantly lower and causes the vehicle to slip and easily get stuck^19^. In addition, even if the tire continues to slip (wheel spin), the frictional force (coefficient) remains low, so the road surface is hardly scraped. Winter tires or tire chains effectively increase the friction between the tire and the road surface when traveling on icy or snow-covered roads. Increasing driving and braking power, they work toward bringing the tire into even contact with the road surface and promoting an “edge effect” to increase the scratching grip. Meanwhile, there is clearly a higher friction coefficient between the ash grains and tires than on icy or compacted snow roads. If ash grains were bonded so that they were stationary, the driving force would depend on the friction coefficient between the tires and the grains, and the vehicle is expected to run with little or no slippage. However, because the ash grains are low in density and move relatively freely, installing tire chains does not help improve running ability because of the greater digging effect of the grains under the driving wheels.

In gravel roads, as in ash-covered roads, crushed stone particles are not bonded to each other, but the two characteristics are widely different. Compared to ash grains, crushed stone particles used for gravel roads generally have an irregular grain size (from a few millimeters to 40 mm), a high flattering ratio, and high density and specific gravity. This makes it more difficult for the particles to be moved by the forces applied by the drive wheels and less likely for the wheels to sink into the layer than ash-covered road surfaces.

### Changes in road conditions with ashfall

Different factors could alter the conditions of ash-covered roads. First, although ashfall thickness increases throughout the eruptive activity, its relative thickness depends on the distance from the vent and the prevailing wind direction, and the grain size also changes. Similar changes apply to ash grains on roads. Furthermore, ash-covered road conditions could be changed secondarily. For example, ruts are formed as vehicles travel the roads, reducing the thickness and changing the shape of grains. Course D was initially built with 30 cm thick ash, but after 7 days and more than 50 running tests, the ash thickness finally shrunk to approximately 20 cm. Also, the depth of the ruts formed after running had clearly decreased as well (Fig. S2). To analyze such changes in road conditions covered in fallen ash, we examined ash grain size and profile using a CAMSIZER P4 (Retsch Technology), a device for measuring grain size and grain shape through dynamic image analysis.

Coarse ash grains collected from test course D did not significantly change in size throughout the test period. Meanwhile, grain size changes were observed in the sample collected from the range of 1 cm thick of ashfall on test course A1 (Fig. S3). The volume ratio of grains with a maximum Feret’s diameter of 2.000–4.000 mm, exceeding 30 vol.%, remained unchanged. In addition, the proportion of grains with a maximum Feret’s diameter exceeding 4.000 mm decreased. Conversely, the proportion of particles with a maximum Feret’s diameter of less than 2.000 mm increased (Fig. S3a, Table S4). This means that larger grains, especially those with a maximum Feret’s diameter exceeding 4.000 mm, were crushed, and the proportion of small grains increased. Grains with maximum Feret’s diameters of 1.000–2.000 mm, 0.500–1.000 mm, and 0.250–0.500 mm with increasing proportions were investigated for their shape. All grain sizes showed a reduction in grain symmetry, roundness, aspect ratio, and convexity. The grain shape change is more pronounced for grains with smaller maximum Feret’s diameters (0.250–0.500 mm) (Fig. S3b–e, Table S5).

Repeated vehicle runs compress the ash road surface and change grain size and shape. These are mainly caused by the force applied by the tires. Compression by load reduces the porosity within the ashfall layer, causing grains to collide and wear away, resulting in large grains changing to finer, more distorted shapes (Fig. S4). The changes in ashfall thickness and rutting depth on course D (Fig. S2) are thought to be due to the compaction of the ashfall layer, reducing porosity between grains and increasing their density, making it more difficult for the tires to sink. Meanwhile, the lack of changes in grain size observed in the coarse ash samples collected from course D could be attributed to the sampling method and amount. The collection method of scooping ash from the test course using a 50 cc polystyrene bottle was considered insufficient to capture grain size variation of the entire layer with ash thicker than 20 cm.

### Factors influencing running-through performance on ash-covered roads

Running-through performance on ash-covered roads mainly depends on the relation between vehicle characteristics and road conditions. Vehicles get stuck on ash-covered roads because the tires of the drive wheels sink into the ashfall layer. The higher the number of drive wheels, the better the driving force distribution, and the lower the likelihood of slips and sinking, and the higher the running ability. Meanwhile, FWD and RWD vehicles distribute a higher driving force per wheel compared with AWD vehicles, causing them to slip and sink more easily into the deposited ash layer. Considering these, the drive system is the most influential factor for running ability among vehicle characteristics, the others being vehicle weight, tire size and tread pattern, motor type, and traction control system. First, the lighter the vehicle weight, the lower the load on the road surface, and tires with a larger contact patch distribute the load and prevent sinking. This study found no correlation between vehicle weight and running distance (Fig. 2), which is attributed to the inverse correlation between vehicle weight and fitted tire size (Table S3). Put simply, when the weight of the vehicle is reduced, the size of the tires fitted tends to become smaller, reducing their ground contact patch. Next, a traction control system can reduce slipping and the probability of getting stuck, but its performance highly depends on the vehicle manufacturer and model. In other words, by developing an optimal traction control system that minimizes the amount of ash grains scraped out in contact with the tread, maximum traction ($F_{trac}$) is ensured even for two-wheel-drive vehicles. Such factors affecting vehicle running ability might also be utilized in the research and development of space exploration vehicles that travel on the surface of extraterrestrial bodies, similar to ash-covered road surfaces.

Among road surface conditions, “thickness of ash” is the most important factor for vehicle running ability on ash-covered roads. Naturally, the thicker the ashfall, the higher the rate at which the drive wheels sink and the higher the probability of getting stuck. FWD and RWD vehicles got stuck when ashfall thickness exceeded approximately 10 cm, while AWD vehicles could drive through even at a thickness of approximately 20 cm (Table 1). Other factors include road slope, wetness, and the grain size of volcanic ash. Road running ability is lower on uphill roads than on flat roads. The grain size and wetness of ash could change ash density and ashfall surface hardness, which might affect running performance, but this must be examined further. For example, on course C2, which was covered with fine-grained ash, when the moisture content was approximately 9% of the ash, the road surface was compacted, with almost no ruts forming even when vehicles passed over it.

The results of the scientific verification of vehicle running ability on ash-covered roads obtained in this running test could help determine appropriate traffic regulations during volcanic eruptions with ashfall. For example, policymakers may consider permitting only AWD vehicles on the road, among other measures, depending on the thickness of ashfall. However, such a policy would exclude the majority of Japanese residents because in most regions, except areas with deep snow, 80% of passenger vehicles owned by residents are two-wheel-drive vehicles^15^. Therefore, rapid disaster recovery and mitigation of economic loss require the immediate removal of ash from the roads to ensure safety and resume road traffic. To achieve this, the national government, local governments, and private companies that manage roads must work toward creating facilities and rules for ash removal.

### Future work

As this study was unable to fully evaluate all vehicle performances on ash-covered roads, future research on the following conditions and/or methods is required.

1. *Braking and turning tests:* These tests are extremely important when examining vehicle driving safety on slippery ash-covered roads. As previously stated, braking and turning tests on the courses were conducted, the results from which will be reported in a subsequent paper.
2. *Vehicle durability tests:* Ash and ash dust can cause various types of damage to vehicles, such as accelerated tire wear, clogged intake filters, and damage to vehicle bodywork, engines, and glass. Therefore, durability evaluations are especially important for disaster prevention organization vehicles that are likely to be used for longer periods on ash-covered roads.
3. *Vehicle conditions:* All vehicles tested in this paper were internal combustion engine vehicles. Compared to internal combustion engines, battery electric vehicles and fuel cell vehicles powered by electric motors, which are becoming more mainstream, allow for finer control over the driving force, which could improve running performances even if they have the same drive systems. However, the weight of battery electric and fuel cell vehicles is heavier than comparable internal combustion engine vehicles, which could affect their dynamic performance, such as braking and turning. As battery electric vehicles do not require air to operate, they may be more durable in dusty environments.
4. *Volcanic ash conditions:* This study tested the three volcanic ash types most common at two active volcanoes, Mt. Fuji, and Sakurajima, each of which have different grain sizes. However, the ash was not fresh because some time had passed since the eruptions or were volcanogenic deposits (see Method for detail). Therefore, the ash may have lost some of its original properties. For example, different from dried coarse-grained volcanic ash, even thin layers of the very fine-grained volcanic ash (less than 1 mm in Feret’s diameter) associated with phreatic eruptions, which contains clay minerals, could easily cause slippages on a road surface^20^ because of the extremely low friction coefficient between the tires and the road surface.

## Methods

Citizen-focused running tests for vehicles and drivers were conducted on the following courses before the workshop.

### Test courses

To reproduce various road surface conditions after ashfall, four types of test courses were prepared: A (flat), B (curve), C (uphill and downhill), and D (hard). All courses were paved and covered with one of three types of volcanic ash with different grain sizes. The grain sizes (maximum Feret’s diameter) were approximately ~ 0.500–16.000 mm, ~ 0.125–8.000 mm, and ~ 0.063–2.000 mm, referred to as coarse-, medium-, and fine-grained ash, respectively (Fig. S5, Table S6). The thickness of ash on the courses varied, ranging from 1 to 20 ~ cm. During the test period, the ash from the test courses was sampled and the moisture content was measured, with the percentage of the coarse-grained ash that covered courses C3 and D being 11–14%.

Running ability tests were mainly conducted on courses A, C, and D (Fig. 1). Course A has four tracks covered with ash of the following grain sizes: A1—coarse, A2—medium, A3—fine, and A4—wet fine. One track is a flat straight line 45 m long, with thickness changing from 1 cm, 5 cm, and 10 cm every 15 m (Fig. 1a). Course C also has a total of four test tracks, which are 30 m long uphill and downhill roads covered with coarse- or fine-grained ash. The gradient was 5%, which is based on the average slope in the urban area of Fujiyoshida City at the northern foot of Mt. Fuji. The climbing performance test was conducted on track C3, which was covered with coarse-grained ash at least 10 cm thick (Fig. 1b). Course D is a 20 m long, flat, and straight road covered with coarse-grained ash over 20 cm thick, which is the toughest for vehicles to run through (Fig. 1c).

### Test methods

Two types of tests were conducted on the course: (1) a start and sudden-start test and (2) a passing test. The former was conducted on courses A and C and sought to evaluate vehicle behavior and stability during start-up. The accelerator openings at start and sudden start were set to 30% or less and full throttle, respectively. The latter was conducted on courses C and D to quantitatively assess the vehicle’s running ability. The vehicles entered the course from a paved road at a speed of 5 km/h and tried to pass through the course while maintaining constant speed. A low-speed setting was selected because of the difficult conditions of driving on ash-covered roads. However, if it became difficult to maintain speed and the vehicle got stuck, the driver immediately took his foot off the accelerator. We measured the running distance from the edge of the course to the front wheels and ash thickness at the point where the wheels were stuck (Fig. 1b, c).

The ruts on the course by running were leveled with rakes every time or were avoided altogether to minimize the influence of changes in ash layer thickness. In addition, the same driver was in charge of almost all tests to minimize errors and habits associated with differences among drivers. No special driving techniques were used in the tests.

### Test vehicles

The production cars used for the tests were nine models commonly used in Japan, each of which has different body types and drive systems (Fig. S6, Table S7). The drive systems included FWD, RWD, and AWD, and vehicle weight ranged from 880 kg to 1600 kg. Except for one vehicle with winter tires, all others had summer tires. Before the test, tire pressure was adjusted to proper values, and the remaining grooves were ensured to be at least 50%. No special modifications were added to the vehicles.

### Supplementary Information

Supplementary Information.

## Data Availability

All data generated during this study are included in this published article and its supplementary information files.

## References

[1] Brown SK, Jenkins SF, Sparks RSJ (2017). Volcanic fatalities database: analysis of volcanic threat with distance and victim classification. J Appl. Volcanol..

[2] EM-DAT. *EM-DAT*: The international disaster database. http://www.emdat.be/Database/Trends/trends.html. Accessed December 2021 (2008).

[3] Suzuki Y (2016). Dynamics of volcanic eruption clouds: Recent progress of numerical models. Bull. Volcanol. Soc. Jpn..

[4] Bonadonna C, Phillips JC, Houghton BF (2005). Modeling tephra sedimentation from a Ruapehu weak plume eruption. J. Geophys. Res..

[5] Suzuki YJ, Koyaguchi T (2009). A three-dimensional numerical simulation of spreading umbrella clouds. J. Geophys. Res..

[6] Working Group on Countermeasures for Wide-Area Ash Falls from Major Volcanic Eruptions. Measures wide-area ash fall from major eruptions—effects of ashfall and countermeasures in the Tokyo metropolitan area—Mt. Fuji eruption as a model case (reports) (2020).

[7] Anyouji N (2007). Estimated damage in the Tokyo Metropolitan Area what will happen in an eruption of Mt. Fuji?. Sci. J. Kagaku.

[8] Yamashita Y, Yamakawa J, Etou R (2015). The effect of volcanic ash to vehicle running motion. Transp. Logist. Conf..

[9] Sakai Y, Nagayoshi S, Kunitomo M (2016). Safety precautions for driving automobiles during field surveys from driving experiments on roads covered with volcanic ash. Civ. Eng. J..

[10] Mt. Fuji Volcano Disaster Prevention Council. Mt. Fuji Hazard Map (Revised) Review Committee Report. *Mt. Fuji Volcano Disaster Prevention Council Secretariat* (2021).

[11] Mt. Fuji Volcano Disaster Prevention Council. Mt. Fuji Hazard Map Review Committee Report. *Mt. Fuji Hazard Map Review Committee Secretariat* (2004).

[12] Tsuya H (1955). Geological and Petrological Studies of Volcano, Fuji, V: 5. On the 1707 eruption of Volcano Fuji. Bull. Earthq. Res. Inst. Univ. Tokyo.

[13] Machida H (1964). Tephrochronological study of volcano Fuji and adjacent areas. J. Geogr..

[14] Miyaji N (1984). Wind Effect on the dispersion of the Fuji 1707 Tephra. Bull. Volcanol. Soc. Jpn..

[15] Japan Automobile Manufacturers Association. Passenger Car Market Trend Survey Report for FY2021. *JAMA* (2022).

[16] Yoshimoto, M., *et al*. Outline of driving test on ashfalls covered roads in Mt. Fuji. JpGU2022, S-VC30-08 (2022).

[17] Mt. Fuji Volcano Disaster Prevent on Council. Mt. Fuji Volcano Wide Area Evacuation Plan. *Mt. Fuji Volcano Disaster Prevention Council* (2023).

[18] Hirao E (2001). Slip of tire, The season why four-wheel drive vehicles are resistant to snowy roads. Mot. Ring.

[19] Onoda M (2004). Skid resistance on road surfaces. Asphalt.

[20] Kiso Town, Nagano. Syntax of referencing in *Mt. Ontake Eruption Disaster Activity Records of 2014*. (ed. General Affairs Division, Kiso Town Hall) 62–63 (Kiso Town, Kiso District, Nagano Prefecture, 2018).

