# Supplementary material for: Evaluation of vehicle running performance on ash-covered roads
Source: Sci Rep. 2023 Dec 6;13:21498. doi: 10.1038/s41598-023-47122-8 (PMC10700594; doi:10.1038/s41598-023-47122-8)
Supplement: Supplementary file 1 — Supplementary Information. [file 41598_2023_47122_MOESM1_ESM.pdf]

“Evaluation of vehicle running performance on ash-covered roads”

Supplementary Information

Tatsuji Nishizawa[1*], Mitsuhiro Yoshimoto[1], Tomohiro Kubo[1], Ryo Honda[1], Setsuya Nakada[2], Nobuko Kametani[1], Yasuhiro Ishimine[1], Shinya Yamamoto[1]

[1] Mount Fuji Research Institute, Yamanashi Prefectural Government

[2]National Research Institute for Earth Science and Disaster Resilience

Supplementary Figures

Fig. S1 to Fig. S6

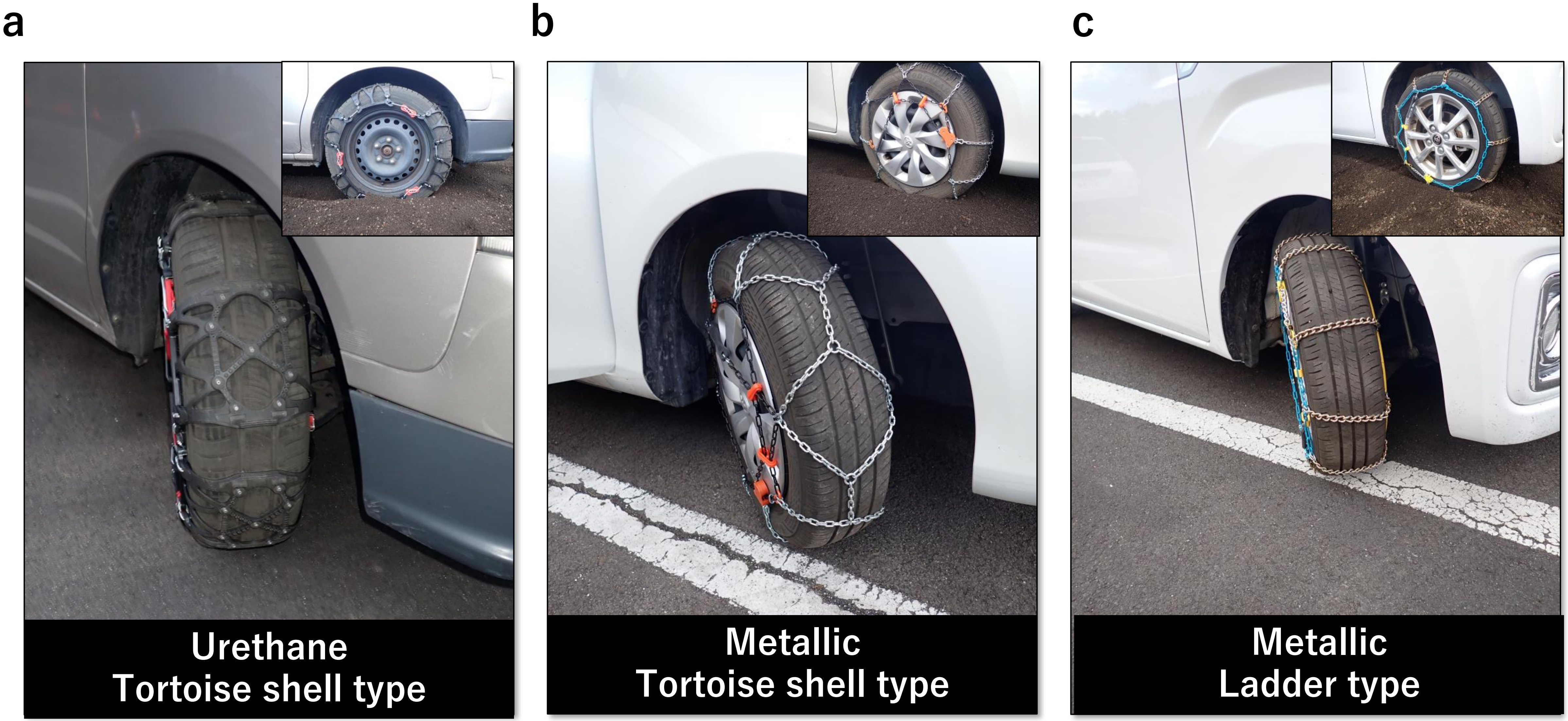

Figure S1. Tire chains installed on the FWD test vehicles. Three types of tire chains with different materials and patterns were used: (a) urethane-tortoise shell, (b) metallic-tortoise shell, and (c) metallic-ladder type.

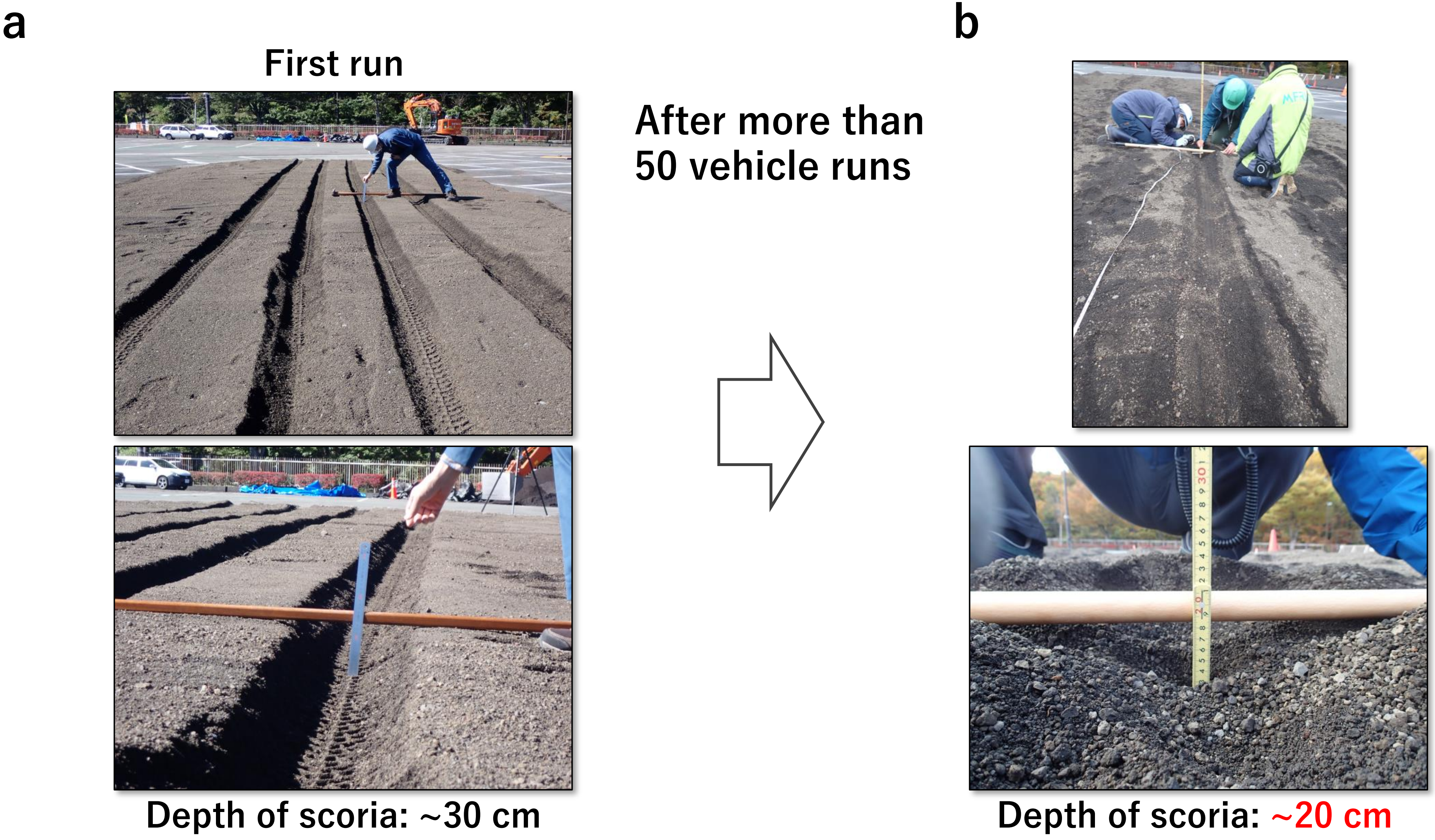
a
First run
Depth of scoria: ~30 cm
After more than 50 vehicle runs
b
Depth of scoria: ~20 cm

Figure S2. Changes in the surface of course D covered with coarse-grained ash. As vehicle runs are repeated, the thickness of ash and the depth of the ruts formed after vehicle running become shallower. (a) First run after construction of the test course: ash thickness was 30 cm. (b) Road surface conditions near the end of the test period after more than 50 instances of repeated running had an ash thickness of approximately 20 cm.

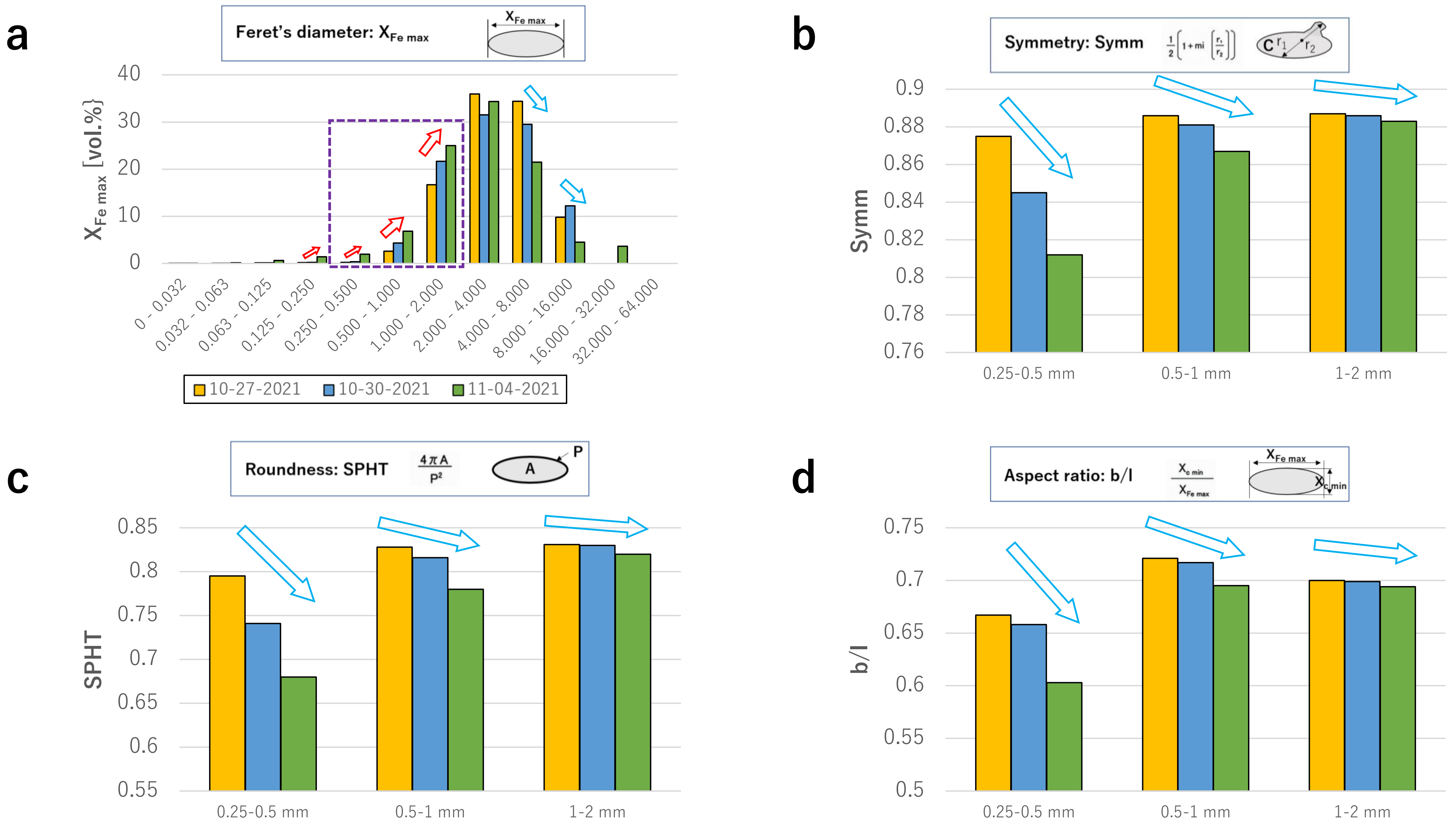
a
Feret's diameter: X Fe max
X Fe max [vol.%]
0 - 0.032
0.032 - 0.063
0.063 - 0.125
0.125 - 0.250
0.250 - 0.500
0.500 - 1.000
1.000 - 2.000
2.000 - 4.000
4.000 - 8.000
8.000 - 16.000
16.000 - 32.000
32.000 - 64.000
10-27-2021
10-30-2021
11-04-2021
b
Symmetry: Symm
Symm
0.25-0.5 mm
0.5-1 mm
1-2 mm
c
Roundness: SPHT
4πA
P²
A
P
SPHT
d
Aspect ratio: b/l
X c min
X Fe max
b/l

Figure S3

e

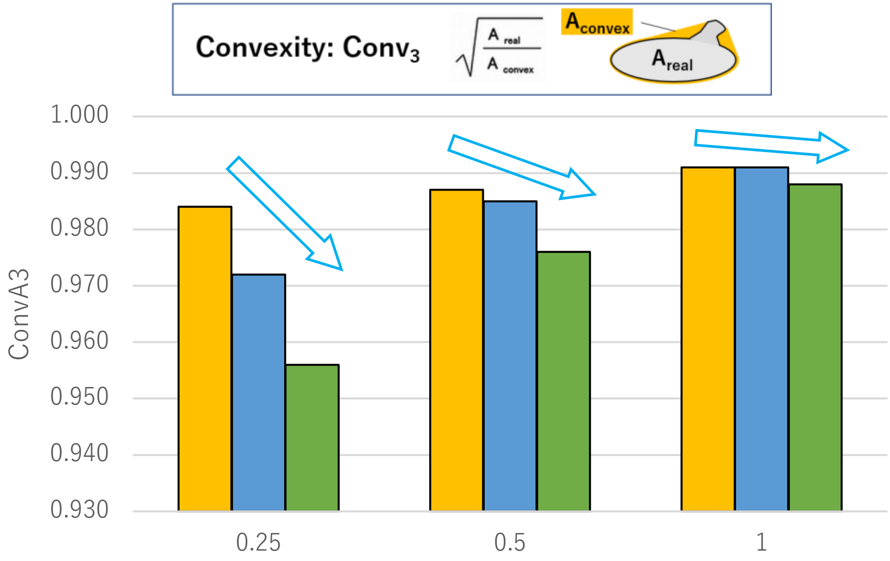

Figure S3. Time variation of the particle size and shape of coarse-grained ash. (a) The volume fraction of particle size based on maximum Feret's diameter. Changes in (b) symmetry, (c) roundness, (d) aspect ratio, and (e) convexity of coarse-grained ash with maximum Feret's diameter from 0.250 mm to 2.000 mm. These were measured using CAMSIZER P4 (Retsch Technology). Data are shown in Tables S4, S5.

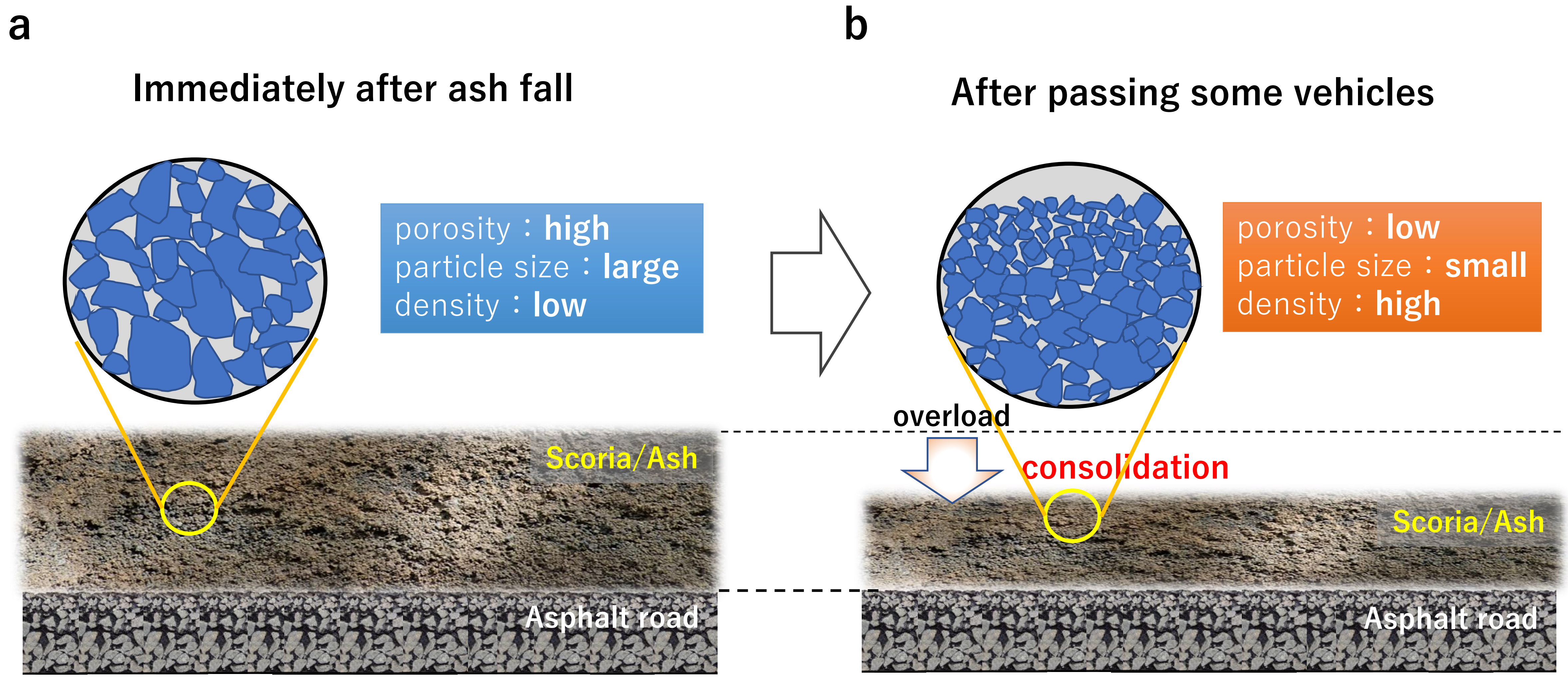

Figure S4. Changes in ash-covered roads. (a) Immediately after ashfall. (b) After repeated vehicle runs. The force that the ash-covered road surface revives from the tires of passing vehicles forms ruts and reduces ashfall thickness. This is attributed to a decrease in porosity, a decrease in grain size of ash, and an increase in density.

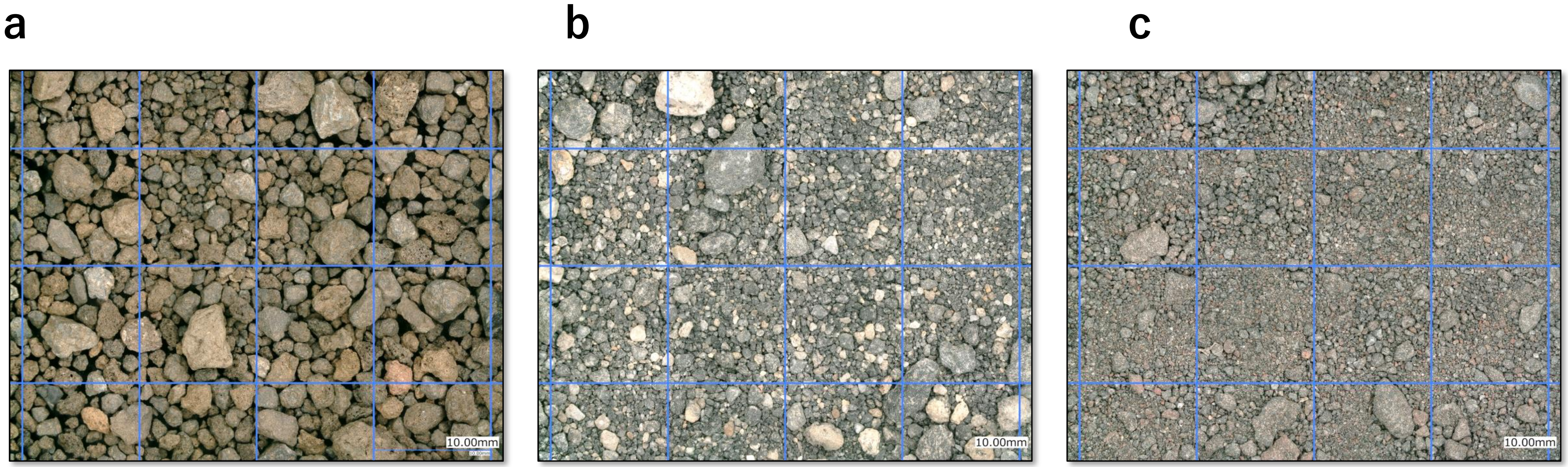

Figure S5. Ash grains used for testing. (a) Coarse-grained ash, (b) medium-grained ash, and (c) fine-grained ash. The blue frame is 4 cm^2. Photos were taken by using a digital microscope (VHX-5000, KEYENCE). See Table S6 for details.

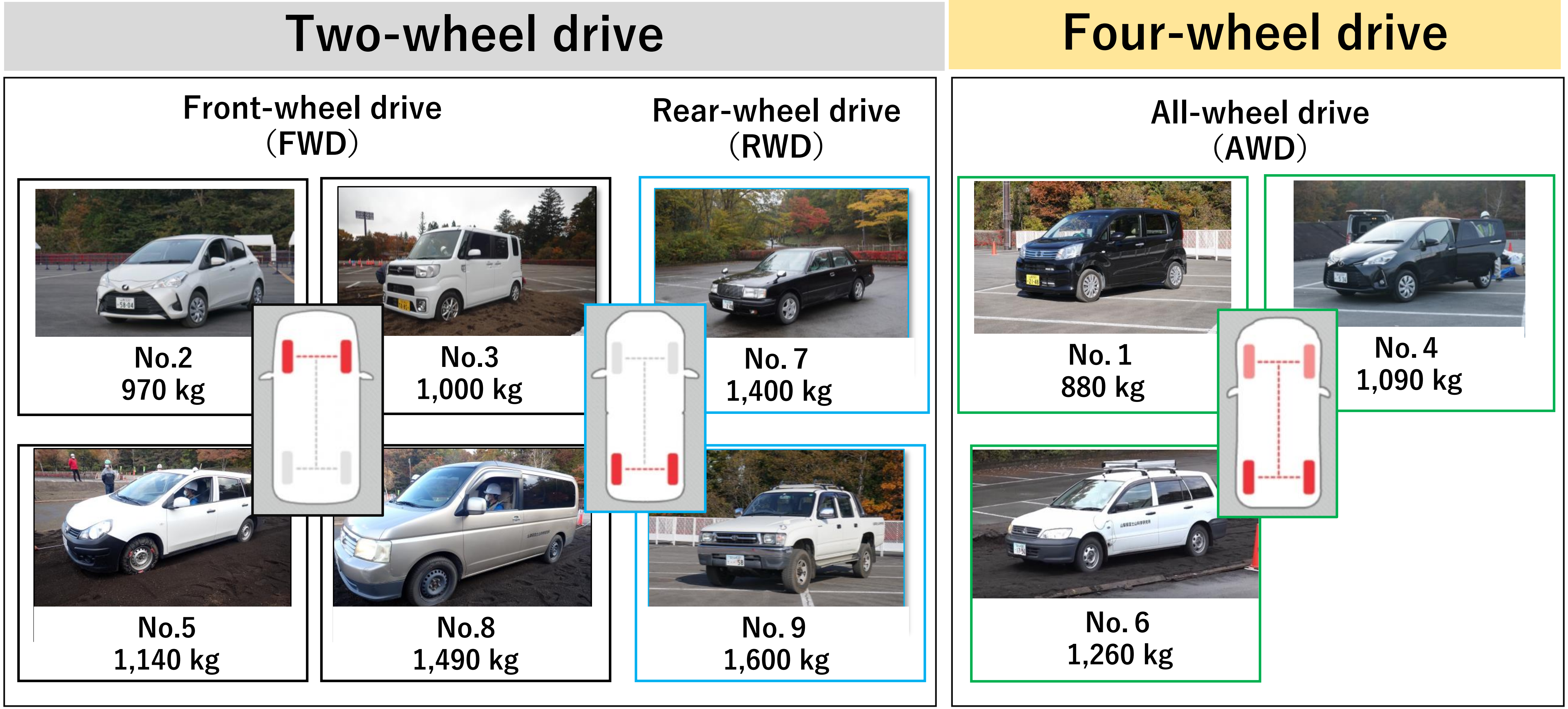

Figure S6. Test vehicles. Nine commercial vehicles with different body types and drive systems: four FWD vehicles, two RWD vehicles, and three AWD vehicles. Vehicle weight ranged from 880 kg to 1,600 kg. Summer tires were installed except for vehicle no. 9. See Table S7 for details.

Supplementary Tables

Table S1 to Table S7

Table S1. **Test courses used for the vehicle dynamic tests.**

Course	A (Flat)				B (Curve)		C (Uphill & Downhill)				D (Hard)
**Course No.**	**A1**	**A2**	**A3**	**A4**	**B1**	**B2**	**C1**	**C2**	**C3**	**C4**	**-**
**Gradient (%)**	0				0	2.5	5				0
**Length (m)**	45				-		30				20
**Radius of curvature (m)**	-				30		-				-
**Thickness (cm)**	1, 5, 10				1		1	~12	~12	1	20~
**Ash grain size**	Coarse	Medium	Fine	Fine (wet)	Fine		Fine		Coarse		Coarse

Table S2. **Results of passing tests on the coarse D, "Hard" (coarse-grain ash, thickness: >20 cm)**

Test vehicle No.	Drive system Vehicle wheght	Test date	Time	Number of tests	Tyre chains	Results	Running distance (m)	Running distance Ave. (m)	Thickness (cm)	Moisture content of ash (%)	Note
No.1	AWD 880kg	11/02/21	13:30	1		Pass	-	-		11	
			13:35	2		Pass	-				
No.2	FWD 970kg	11/01/21	16:02	1		Stuck	18.99	11.3	16	11	*1
				2		Stuck	9.63		18.5		*1
				3		Stuck	5.74		18.5		*1
				4		Stuck	5.94		16		*1
				5		Stuck	14.43		17.5		*1
			16:48	6		Stuck	12.88		22		*2
		11/02/21	15:32	1C	Metallic Tortoise shell type	Pass	-	10.8		11	
			15:33	2C		Stuck	10.08		21		
			15:37	3C		Pass	-				*3
			15:38	4C		Stuck	11.61		21		*3
No.3	FWD 1,000kg	11/01/21	15:45	1		Stuck	4.76	7.2	14.0	11	
				2		Stuck	4.65		14.5		*4
				3		Stuck	11.50		17.0		
				4		Stuck	5.36		14.5		
			16:00	5		Stuck	9.56		16.0		
		11/02/21	15:25	1C	Metallic-Lader type	Stuck	4.26	5.1	17	11	
			15:25	2C		Stuck	6.25		23		
			15:25	3C		Stuck	4.69		19		
No.4	FWD/AWD 1,090kg	11/02/21	13:31	1		Stuck[*5]	10.83	-	28	11	*5
			13:36	2		Pass	-	-			
			13:39	3		Pass	-				
No.5	FWD 1,140kg	10/28/21	11:47	1		Stuck	4.39	5.2	20.5	12	
			11:56	2		Stuck	4.32		16		
			12:02	3		Stuck	5.52		21		
			12:06	4		Stuck	6.42		23.5		
		11/02/21	15:42	1C	Urethane Tortoise shell type	Stuck	6	5.8	18	11	
			15:45	2C		Stuck	5.59		20.5		
No.6	AWD 1,260kg	10/29/22	14:41	1		Pass	-	-		14	*3
			14:45	2		Pass[*6]	-				*3, 6
No.7	RWD 1,400kg	11/02/21	11:26	1		Stuck	10.62	12.1	19	11	
			11:30	2		Stuck	12.52		19		
			11:33	3		Stuck	9.76		19		
			11:36	4		Stuck	15.60		20		
			11:42	5		Pass	-				*7
			11:44	6		Pass	-				*7
No.8	FWD 1,490kg	11/02/21	13:18	1		Stuck	17.20	12.4	23	11	
			13:24	2		Pass	-		23		
			13:25	3		Stuck	7.63		23		*8
		11/02/21	15:47	1C	Urethane Tortoise shell type	Stuck	17.49	15.4	21		
			15:50	2C		Stuck	13.25		19		

FWD or RWD	Colorless cells are two-wheel drive vehicles or modes.
AWD	Gray cells are four-wheel drive vehicles or modes.

[*1]Traction Control system was ON

[*2]Traction Control system was OFF

[*3] It was driven by a different person than the main test driver.

[*4]The vehicle's tires got into the ruts even though the driver tried to avoid the ruts when entering the course

[*5] The AWD mode was turned off, so the vehicle was in FWD.

[*6] Driving test with repeated starting and stopping. The depth of the ruts were 8 to 9 cm.

[*7]The drive wheels were slightly slipped.

[*8]The vehicle's tires got in the the ruts around 6m from the entrance of the test course.

Table S3. Results of running performance tests on the course C3, "5% uphill" (Coarse-grain ash, thickness: ~12 cm).

Test vehicle No. Drive system Vehicle wheght		Test date and time		Number of tests	Tyre chains	Results	Running distance (m)	Running distance Ave. (m)	Thickness (cm)	Moisture content of ash (%)	Note
No.1	AWD 880kg	11/02/21	14:05	1		Pass	-	-	-	13	
No.2	FWD 971kg	11/02/21	13:56	1		Stuck	7.32	**7.3**	11	13	
			15:00	1C	Metallic Tortoise shell type	Stuck	5.43	**5.4**	12.5		
No.3	FWD 1,000kg	11/02/21	13:52	1		Stuck	5.97	**6.0**	13	13	
			14:53	1C	Metallic-Lader type	Stuck	3.63	**3.7**	14		
			14:57	2C		Stuck	3.69		12.5		
No.4	FWD/AWD 1,090kg	11/02/21	14:08	1		Stuck	7.98	-	9	13	*1
			14:10	2		Pass	-	-	-		
No.5	FWD 1,140kg	11/02/21	14:00	1		Stuck	6.73	**6.7**	11	13	
			15:03	1C	Urethane Tortoise shell type	Stuck	5.13	**5.1**	11		
			15:06	2C		Stuck	11*2	-	13.99		*2
No.6	AWD 1,260kg	10/31/21	15:25	1		Pass	-	-		13	*3, 4
				2		Pass	-				*3, 5
			15:30	3		Pass	-				*3
No.8	FWD 1,490kg	11/02/21	14:02	1		Stuck	11.23	**11.2**	13	13	
			15:09	1C	Urethane Tortoise shell type	Stuck	9.00	**9.0**	8.04		
			15:12	2C		Pass*3	-	-			*6
No.9	RWD/AWD 1,600kg	11/02/21	14:15	1		Stuck	7.24	**7.3**	10.5	13	*7
			14:17	2		Stuck	7.44		11		*8
			14:22	3		Pass	-	-	-		*9

FWD or RWD	Colorless cells are two-wheel drive vehicles or modes.
AWD	Gray cells are four-wheel drive vehicles or modes.

*1 The AWD mode was turned off, so the vehicle was in FWD.

*2 Passing at a speed of 10 to 20 km/h with the accelerator pedal somewhat open.

*3 The test course surface was wet due to the rainy weather. The moisture content of the scoria was 13%.

*4 Driving test with repeated starting and stopping.

*5 Driving test with repeated sudden starting and stopping. The all four wheels did not slip during the sudden acceleration, but all wheels bounced and the vehicle body shook.

*6 After the vehicle getting stuck, move backward slightly and start again. After a total of 14 repetitions of backward and forward, the test vehicle passed through the uphill course.

*7 The vehicle was in "2H" mode; the transfer case was off.

*8 The vehicle was in "2H" mode and rear-differential gear was locked.

*9 The vehicle was in "4H" mode and rear-differential gear was opned.

Table S4. Volume fraction of maximum Feret diameter of the coarse-grain ash particles on the course A1.

**Grain size**		Coarse		
**Scoria/Ash**		Scoria		
**Volcano**		Mt. Fuji		
**Source**		The 1707 Houei eruption		
**Sampling course**		A1 [depth: 1cm]		
**Sampling date**		10-27-2021	10-30-2021	11-04-2021
**Volume fraction of the maximum Feret diameter[*1] $X_{Fe\ max}$ [mm]**	<0.032	0.005	0.004	0.035
	0.032-0.063	0.030	0.024	0.110
	0.063-0.125	0.133	0.130	0.612
	0.125-0.250	0.169	0.214	1.423
	**0.250-0.500**	**0.202**	**0.360**	**1.967**
	**0.500-1.000**	**2.586**	**4.321**	**6.840**
	**1.000-2.000**	**16.690**	**21.694**	**24.997**
	2.000-4.000	35.949	31.536	34.358
	4.000-8.000	34.425	29.501	21.497
	8.000-16.000	9.811	12.216	4.521
	16.000-32.000	0.000	0.000	3.640
	32.000-64.000	0.000	0.000	0.000
	>64.000	0.000	0.000	0.000

[*1] Particle size distributions were measured using CAMSIZER P4 (Retsch Technology GmbH) after drying.
Particle profiles for bold Feret diameter are shown in Table S6.
Tables is colored by the value of the maximum Feret diameter on each sampling date.

Table S5. Change in particle profile of the coarse-grain ash particles with maxium Feret diameter of 0.250-2.000 mm.

**Grain size**		Coarse		
**Scoria/Ash**		Scoria		
**Volcano**		Mt. Fuji		
**Source**		The 1707 Houei eruption		
**Sampling course**		A1 [depth: 1cm]		
**Sampling date**		10-27-2021	10-30-2021	11-04-2021
**Volume fraction of the maximum Feret diameter*1 $X_{Fe\,max}$ [mm]**	0.250-0.500	0.202	0.360	1.967
	0.500-1.000	2.586	4.321	6.840
	1.000-2.000	16.690	21.694	24.997
**Average of Roundness*1 [SPHT] at each X_{Femax}**	0.250-0.500	0.795	0.741	0.680
	0.500-1.000	0.828	0.816	0.780
	1.000-2.000	0.831	0.830	0.820
**Average of Symmetry*1 [Symm] at each X_{Femax}**	0.250-0.500	0.875	0.845	0.812
	0.500-1.000	0.886	0.881	0.867
	1.000-2.000	0.887	0.886	0.883
**Average of Aspect ratio*1 [b/l] at each X_{Femax}**	0.250-0.500	0.667	0.658	0.603
	0.500-1.000	0.721	0.717	0.695
	1.000-2.000	0.700	0.699	0.694
**Average of Convexity*1 [$Conv_3$] at each X_{Femax}**	0.250-0.500	0.984	0.972	0.956
	0.500-1.000	0.987	0.985	0.976
	1.000-2.000	0.991	0.991	0.988

*1 Particle size and profiles were measured using CAMSIZER P4 (Retsch Technology GmbH) after drying.
Tables is colored according to its value in each diameter.

Table S6. Volume fraction of maximum Feret diameter of the Coarse-, Medium-, and Fine-grain ash particls used for the test courses.

Grain size		Coarse	Medium	Fine
Scoria/Ash		Scoria	Ash	Ash
Volcano		Mt. Fuji	Sakurajima	Mt. Fuji
Source		The Hoei eruption in 1707 AD.	Alluvial fan deposits at the foot of volcano[*1] (Osumi River).	Alluvial fan deposits at the western foot of volcano[*1] (Osawa).
Volume fraction of the maximum Feret diameter[*2] $X_{Fe\ max}$ [mm]	<0.032	0.009	0.010	0.026
	0.032-0.063	0.174	0.245	0.871
	0.063-0.125	0.747	1.762	7.451
	0.125-0.250	0.737	7.586	28.408
	0.250-0.500	1.069	21.072	30.476
	0.500-1.000	6.652	28.104	15.552
	1.000-2.000	23.318	21.598	8.442
	2.000-4.000	35.270	12.296	4.436
	4.000-8.000	22.822	6.743	3.576
	8.000-16.000	9.202	0.584	0.762
	16.000-32.000	0.000	0.000	0.000
	32.000-64.000	0.000	0.000	0.000
	>64.000	0.000	0.000	0.000
Sampling course		A1	A2	A3
Sampling date		10-20-2021	10-20-2021	10-20-2021
Water content [%]		18	4	7

[*1] Volcanogenic deposits.

[*2] Particle size distributions were measured using CAMSIZER P4 (Retsch Technology GmbH) after drying.

Tables is colored by the value of the maximum Feret diameter on each sampling date.

Table S7. **Test vehicles list.**

Vehicle No.	No.1	No.2	No.3	No.4	No.5	No.6	No.7	No.8	No.9
Body type	Tall Wagon	Compact car	Hight-wagon	Compact car	Station Wagon	Station Wagon	Sedan	Minivan	Pik-up truck
Vehicle weight (kg)	880	970	1,000	1,090	1,140	1,260	1,400	1,490	1,600
Front axle weight (kg) & weight distribution (%)	530 (60)	600 (62)	580 (58)	680 (62)	670 (59)	740 (59)	750 (54)	840 (56)	940 (59)
Rear axle weight (kg) & weight distribution (%)	350 (40)	370 (38)	420 (42)	410 (38)	470 (41)	520 (41)	650 (46)	650 (44)	660 (41)
Drive system	AWD	FWD	FWD	FWD/AWD	FWD	AWD	RWD	FWD	RWD/AWD
Four-wheel drive system	Automatic/Passive on-demand (viscous coupling)	-	-	Automatic/ Active on-demand (electronic controlled cupling)	-	Full time	-	-	Part time with rear differential lock function
Tire	Summer	Summer	Summer	Summer	Summer	Summer	Summer	Summer	Winter/ Studless
Tire size	155/65R14	165/70R14	155/65R14	165/70R14	165/80R13	165/80R13	195/70R14	195/65R15	215/80R15
Tire Chain	-	Metallic Tortoise shell	Metallic Ladder	-	Urethane Tortoise shell	-	-	Urethane Tortoise shell	-
Traction Controll System	-	Yes	Yes	Yes	-	-	-	-	-
Model year	2019	2018	2021	2019	2016	2007	1997	2001	1998
Displacement(L)/ Horsepower(HP)	0.65/52	0.99/69	0.65/52	1.32/95	1.49/82	1.46/90	1.98/135	1.99/160	1.99/110
